# Effectiveness of tongue‐tie assessment tools in diagnosing and fulfilling lingual frenectomy criteria: a systematic review

**DOI:** 10.1111/adj.12921

**Published:** 2022-07-04

**Authors:** A Hatami, CW Dreyer, MJ Meade, S Kaur

**Affiliations:** ^1^ Adelaide Dental School, Faculty of Health and Medical Sciences The University of Adelaide Adelaide Australia

**Keywords:** Ankyloglossia, frenoluplasty, frenotomy, tongue tie

## Abstract

It is unclear how effective tongue‐tie classification assessment tools are in diagnosing symptomatic tongue‐tie and fulfilling lingual frenectomy criteria. The purpose of this systematic review is to determine and evaluate any association between tongue‐tie severity, as measured by pre‐treatment assessment tools, and post‐operative outcome following tongue‐tie division. PubMed, EMBASE, and the Cochrane search engines were used to retrieve articles published between 1947 and 2021. Included studies consisted of patients with symptomatic tongue‐tie, assessment by either the Coryllos, Kotlow, or Hazelbaker Assessment Tool for Lingual Frenulum Function (HATLFF) classification tool, and tongue‐tie division. A total of 205 abstracts were identified; 31 studies met the criteria for a full‐text review, of which, only 14 studies met the criteria for data extraction and analysis. Six studies used the HATLFF, 2 studies used the Kotlow, 5 studies used the Coryllos, and 1 study used a combination of both Kotlow and Coryllos methods. Significant heterogeneity was evident across all studies. No statistical correlation between the two variables could be determined. Although tongue‐tie division procedures appear to provide benefits in breastfeeding and speech, there are no data to suggest a statistically significant association between the severity of tongue‐tie, and the correct identification of patients who would benefit from tongue‐tie division. © 2022 Australian Dental Association.

**Abbreviations and acronyms**
HATLFF: Hazelbaker Assessment Tool for Lingual Frenulum Function
IBFAT: infant breastfeeding assessment tool
LATCH: latch, audible, nipple type, comfort and help
RCT: randomized controlled trial
U‐TAP: Urimal test of articulation and phonation
VAS: Visual Analogue Scale

## INTRODUCTION

Ankyloglossia, commonly referred to as tongue‐tie, is a common congenital condition of the sublingual frenulum characterized by a functional limitation of the tongue. It is often associated with breastfeeding difficulties and can be surgically managed by either frenotomy (also known as frenulotomy), in which the frenulum attachment is surgically relocated, or frenectomy (also known as frenuplasty), in which the frenulum and its attachment are completely removed.^1^

Despite recent publications showing a dramatic increase in the number of articles associated with lingual frenectomy and tongue‐tie,^2^ there is disagreement over interventions. Some advocate prompt surgical management upon recognition to improve breastfeeding. However, not all clinical cases of tongue‐tie require intervention as most resolve with time and caution has been recommended.^3^

The above inconsistency is partly due to variations in diagnosis.^4^ Moreover, it is unclear from the available body of evidence whether the current classification systems, i.e., Coryllos, Kotlow and the Hazelbaker Assessment Tool for Lingual Frenulum Function (HATLFF), can identify those patients with symptomatic tongue‐tie who would benefit from lingual frenectomy.

Considering these shortcomings, a systematic review was conducted to identify a possible significant statistical correlation between pre‐treatment severity scores of tongue‐tie and post‐treatment outcomes in breastfeeding and speech.

## MATERIALS AND METHODS

### Protocol

**Fig. 1:**
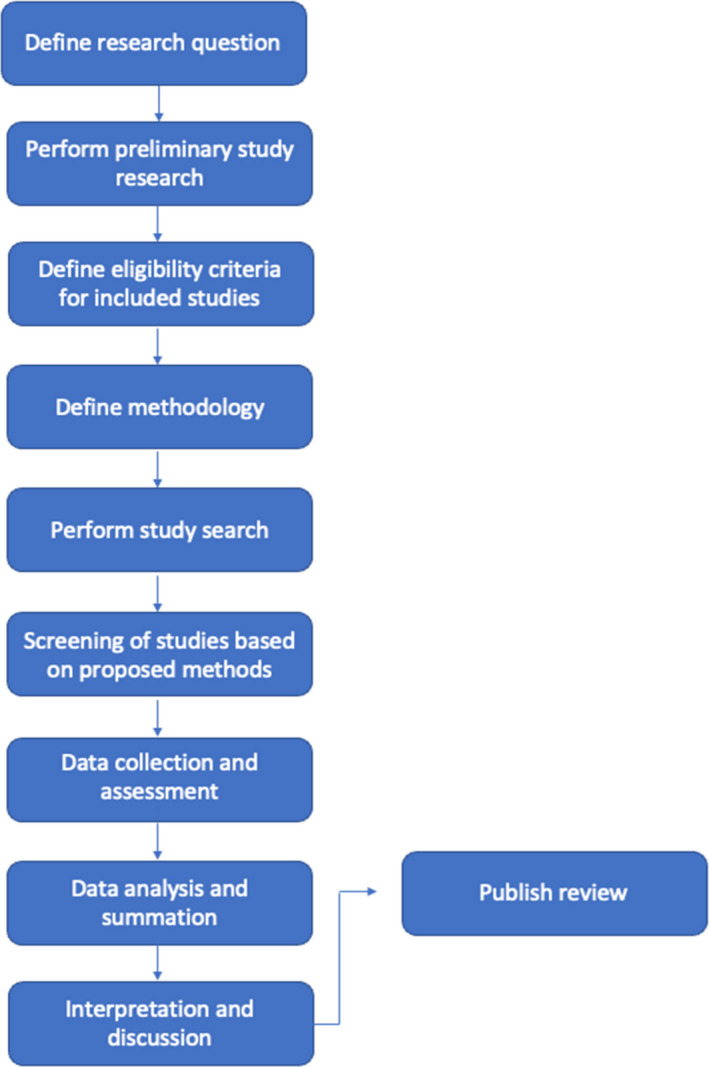
Stages of protocol design. [Colour figure can be viewed at wileyonlinelibrary.com]

The current review adhered to the preferred reporting items for systematic review and meta‐analysis protocol (PRISMA‐P).^5^ A detailed research proposal and protocol was designed for the purpose of identifying the study scope, objectives, aims and methodology (Fig. 1). Data collection checklists were obtained from the Joanna Brigg’s critical appraisal tools and were used to assess the quality of included studies.^6^

### Eligibility criteria

The population under study included symptomatic paediatric patients identified with tongue‐tie who had undergone surgical intervention by lingual division (frenotomy or frenectomy). Tongue‐tie classification assessment tools for diagnosing symptomatic and lingual frenectomy criteria (Coryllos, Kotlow and Hazelbaker) were compared relative to their ability to correctly identify improvements in breastfeeding and/or speech.^7^

Studies were required to be either observational or interventional, and to adhere to level 4 (or above) according to the criteria defined by the Oxford Centre for Evidence‐Based Medicine.^8^ Only articles in the English language were included, and duplicate studies were excluded.

### Information sources

**Fig. 2:**
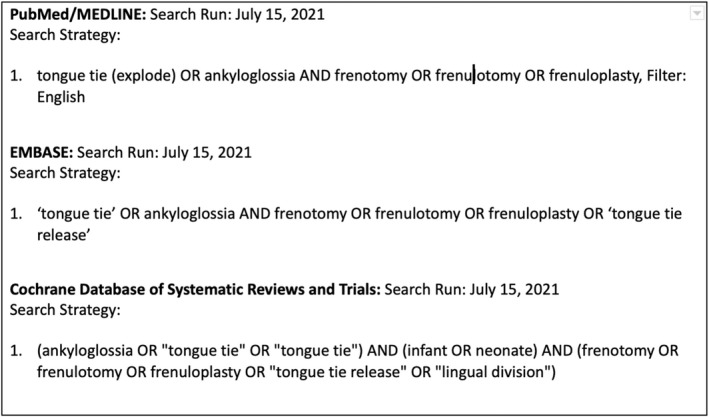
Details of literature search strategy.

A comprehensive search strategy was used to search the following databases: PubMed/MEDLINE, EMBASE, and the Cochrane Database of Systematic Reviews and Trials (Fig. 2). Titles and abstracts were obtained for all studies identified by the search strategy.

### Study selection

Two authors independently reviewed the list of article titles and abstracts generated by the comprehensive search strategy for any studies that met the criteria. A third, more senior author was involved during any selection conflict. Subsequently, full‐text articles were in‐depth evaluated in relation to the inclusion criteria.

The authors assessed the quality of the studies using the Joanna Brigg’s critical appraisal tool kit. This included several checklists that determined the inclusion or exclusion of case reports, case series, cohort studies, randomized controlled trials (RCT) studies and systematic reviews.^6^

### Data extraction

The review authors independently extracted data from full‐text articles using a pre‐designed tabulated Excel (Microsoft Corporation, Washington, USA) spreadsheet to manage the information. The following data were extracted: study design, number of subjects, age of participants, description of intervention (frenectomy or no frenectomy), description of classification system (Coryllos, Kotlow or Hazelbaker), outcome measures (breastfeeding and speech) and the reported statistical effect estimate along with *P*‐value if available.

### Synthesis of results

A descriptive approach was undertaken to report the results of this systematic review. Data were synthesized into tables, according to the nature of the pre‐treatment classification assessment tool. No meta‐analysis was performed due to the heterogeneity of the data and outcome measures.

### Assessment of risk of bias in included studies

The review authors independently assessed the risk of bias of the included study based on the criteria documented in the Cochrane Handbook for Systematic Reviews Interventions for RCTs studies.^9^ The authors gave particular attention to the following items: (i) sample collection; (ii) inclusion and exclusion criteria; (iii) validated classification tools; (iv) postoperative outcome measures, (v) pre‐treatment severity scores. Each study was marked as ‘low’, ‘unclear’ or a ‘high’ risk of bias.

An assessment for non‐RCT studies was performed using the Methodological Index for Non‐Randomised Studies (MINORS).^10^ This validated assessment tool is designed specifically for non‐RCT studies using 12 items, each of which is scored as 0 (not reported), 1 (reported but inadequate) and 2 (reported and adequate). The tool also differentiates between comparative and non‐comparative studies, allocating the first 8 items (16 points in total) to the former, and an additional 4 items (24 points in total) to the latter, with an ideal scoring value near the maximum possible points.

## RESULTS

### Study selection

**Fig. 3:**
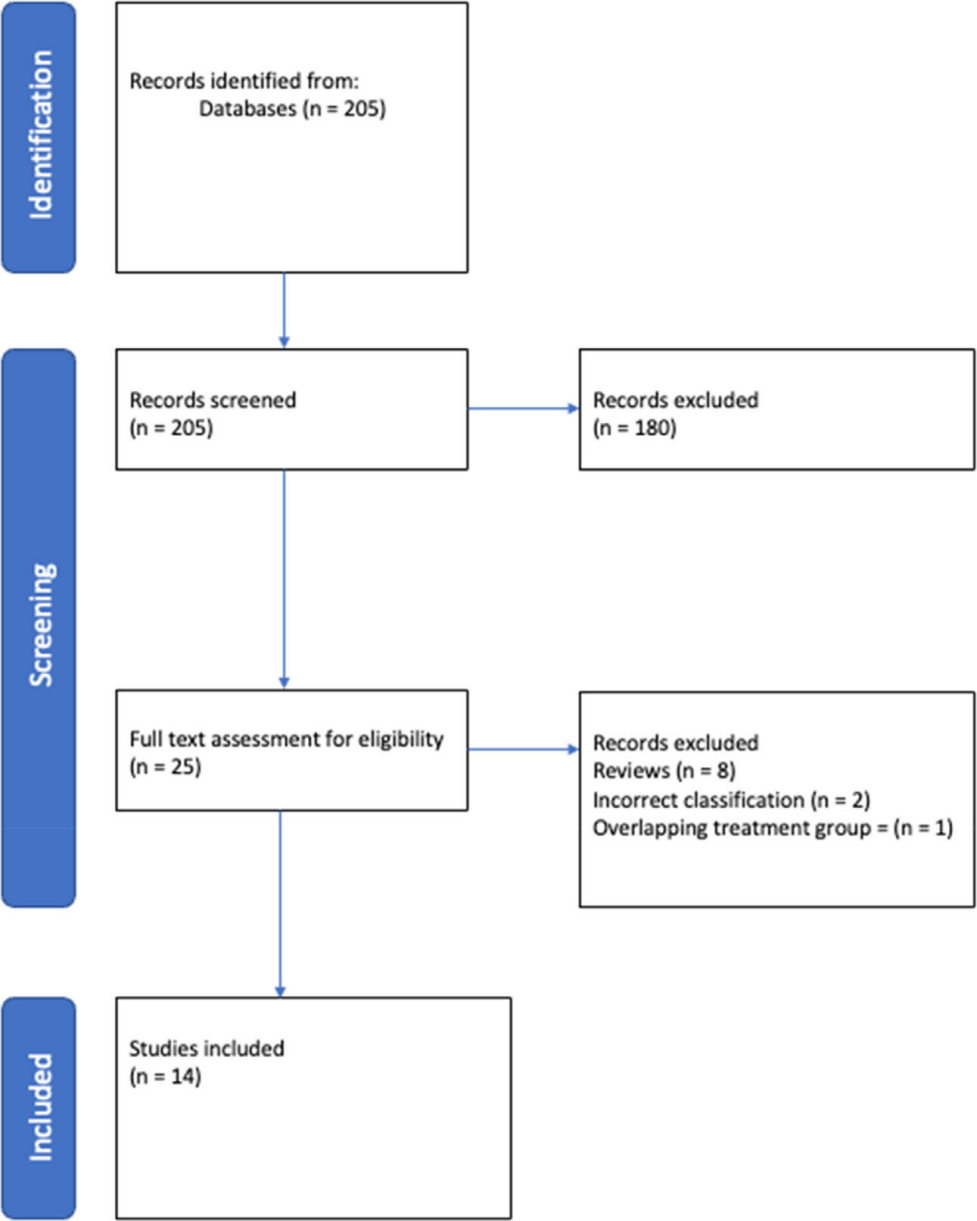
Flow diagram of article selection process. [Colour figure can be viewed at wileyonlinelibrary.com]

A total of 205 abstracts were extracted from the databases. The search and exclusion process are illustrated in Fig. 3. A total of 25 studies met the criteria for full‐text review, and the remaining 180 were eliminated because there was no discussion of tongue‐tie, tongue‐tie surgery or one of the mentioned classification systems (Coryllos, Kotlow or Hazelbaker) described in detail within Table 1.

**Table 1:** Pre‐treatment assessment of tongue‐tie

HATLFF grading system	Coryllos grading system	Kotlow grading system
If 24 points = normal	Type I: AoF to the tip of the tongue	Class I: AoF 12–16 mm from tip of the tongue
If 14 points (functional) = normal (regardless of appearance score)	Type II: AoF 2–4 mm from tongue tip	Class II: AoF 8–11 mm from tip of the tongue
If 11 (functional) and 10 (appearance) = acceptable	Type III: AoF to the mid‐tongue	Class III: AoF 4–7 mm from tip of the tongue
If <11 (functional) and/or <8 (appearance) score = functionally and/or anatomically impaired	Type IV: AoF against the base of tongue	Class IV: AoF 0–3 mm from tip of the tongue

AoF: Attachment of Frenulum.

Coryllos: Type I (mild), Type II (moderate), Types III & IV (severe) tongue‐tie. Kotlow: Class I (mild), Class II (moderate), Class III (severe), Type IV (complete) tongue‐tie.

After further review, 14 studies met the criteria for data extraction and analysis (Table 3)^11^, ^12^, ^13^, ^14^, ^15^, ^16^, ^17^, ^18^, ^19^, ^20^, ^21^, ^22^, ^23^ and 11 were excluded for the following reasons: 8 were reviews, 2 used classification systems not related to the current review and 1 had overlapping treatment groups (Table 2).^24^, ^25^, ^26^, ^27^, ^28^, ^29^, ^30^, ^31^, ^32^, ^33^

**Table 2:** Characteristics of studies excluded in the systematic review

Study	Study design	Reason for exclusion
O'Shea JE *et al*. (2017)^24^	Systematic review	Study conducted was a systematic review
Walsh J and Tunkel D (2017)^25^	Review	Study conducted was a review
Power RF and Murphy JF (2015)^26^	Review	Study conducted was a review
Ingram J and Johnson D (2016)^27^	Observational	Classification system not related to current review
Nakhash R, *et al*. (2019)^28^	Systematic review	Study conducted was a systematic review
Diercks GR, *et al*. (2020)^29^	Observational	Overlapping treatment groups
Kotlow LA (2013)^31^	Review	Study conducted was a review
Hentschel R (2018)^30^	Review	Study conducted was a review
Kotlow L (2011)^39^	Review	Study conducted was a review
Shekher R, *et al*. (2021)^32^	Review	Study conducted was a review
Emond A, *et al*. (2014)^33^	RCT	Classification system not related to current review

RCT = randomized controlled trial.

### Study characteristics

The study design, intervention type, and classification tools used in the 14 studies are shown in Table 3. There were a total of 1145 subjects in the 14 studies. Of the 1145 subjects, 921 underwent ‘frenotomy’, 167 ‘frenuloplasty’, 55 ‘frenulotomy’ and 2 ‘frenectomy’.

**Table 3:** Characteristics of studies included in the systematic review

Study	Study quality	Study design	N	Age of participants	Intervention type	Classification	Outcome
Amir LH, *et al*. (2005)^11^	6*	Case series	35	3–98 days	Frenotomy	HATLFF	Breastfeeding
Ballard JL, *et al*. (2002)^12^	8*	Case series	123	N/A	Frenuloplasty	HATLFF	Breastfeeding
Belmehdi A, *et al*. (2018)^13^	6*	Case series	2	15 & 13 years	Frenectomy	Kotlow	Speech
Bundogji N, *et al*. (2020)^14^	10*	Prospective	133	1–33 days	Frenotomy	Coryllos	Breastfeeding
Buryk M, *et al*. (2011)^15^	High risk^†^	RCT	30	1–5 weeks	Frenotomy	HATLFF	Breastfeeding
Ferrés‐Amat E, *et al*. (2017)^16^	12*	Descriptive	88	0–26 weeks	Frenotomy	Coryllos	Breastfeeding
Ghaheri BA, *et al*. (2017)^17^	12*	Prospective	237	0–12 weeks	Frenotomy	Coryllos, Kotlow	Breastfeeding
Jamani NA, *et al*. (2020)^18^	6*	Case series	3	2–12 weeks	Frenotomy	HATLFF	Breastfeeding
Kim TH, *et al*. (2020)^19^	High risk^†^	RCT	37	3–7 years	Frenotomy and Frenuloplasty	Kotlow	Speech
O'Callahan C, *et al*. (2013)^20^	13*	Prospective	299	0–46 weeks	Frenotomy	Coryllos	Breastfeeding
Schlatter SM, *et al*. (2019)^21^	10*	Observational	30	N/A	Frenulotomy	HATLFF	Breastfeeding
Steehler MW, *et al*. (2012)^22^	11*	Retrospective	78	2.5 weeks (mean)	Frenotomy	Coryllos	Breastfeeding
Yousefi J, *et al*. (2015)^23^	High risk^†^	RCT	50	<12 years	Frenulotomy and frenuloplasty	HATLFF	Breastfeeding

HATLFF = Hazelbaker Assessment Tool for Lingual Frenulum Function.

* MINORS: Methodological Index for Non‐Randomised Studies, a 12‐item assessment tool with a maximum of 16 points for comparative studies and 24 points for non‐comparative studies. Ideal scoring near the maximum points possible.

^†^ Cochrane Handbook for Systematic Reviews Interventions for randomized controlled trials (RCT) studies. Assessment items were pre‐defined by authors and articles were labelled ‘low’, ‘unclear’ or ‘high’ risk of bias accordingly.

### Studies with breastfeeding outcomes

Of the 11 studies that reported breastfeeding outcomes (Table 3), 9 were subjective reports and 2 were validated assessment reports. Schlatter *et al*. (2019) compared pre‐ and post‐division latch, audible, nipple type, comfort and help (LATCH) scores in cohorts with low (HATLFF) functional and appearance mean scores of 11.6 ± 2.1 and 6.5 ± 2.3, respectively, and demonstrated improved mean scores from 7.3 ± 1.9 to 9.3 ± 0.9.^21^ Buryk *et al*. (2011) compared pre‐ and post‐division infant breastfeeding assessment tool (IBFAT) scores in cohorts with low HATLFF functional and appearance mean scores of 9.4 ± 2.6 and 6.0 ± 1.6, respectively, and significantly demonstrated (*P* = 0.029) improved mean scores from 9.3 ± 0.69 to 11.6 ± 0.81.^15^ The HATLFF score has a maximum score of 24 points (functional: 14 points, anatomical: 10 points), with scores below 11 for function and 8 for appearance indicating tongue‐tie.^34^

All studies describing subjective outcomes reported improvements in post‐intervention breastfeeding. Amir *et al*. (2005) reported low HATLFF functional and appearance mean scores of 10.9 ± 0.57 and 5.9 ± 1.5, respectively, followed by a subjective outcome report of 83% improvement in breastfeeding and 17% reporting no difference. The authors also noted no change in HATLFF scores post‐surgery.^11^ Yousefi *et al*. (2015) only reported mean appearance scores of the HATLFF in two groups undergoing different lingual division techniques (6.2 ± 0.24 and 6.0 ± 0.20). The authors used a subjective scale consisting of the following rank: 0 = no change, 1 = improved, 2 = good improved, 3 = full resolution. An improvement in breastfeeding post‐intervention was reported in both groups (2.61 ± 0.52 and 2.33 ± 0.50).^23^ O’Callahan *et al*. (2013) used the Coryllos classification system with the following patient characteristics: Types 1–2 (n = 46), Type 3 (n = 107) and Type 4 (n = 146). Breastfeeding outcomes were subjectively reported with regards to nipple pain, in which pre‐treatment questionnaires reported 146 mothers complained of nipple pain, and the post‐division assessment resulted in resolution for 71 mothers (49%), with 75 mothers (51%) still experiencing nipple pain.^20^ Similarly, Bundogji *et al*. (2020) also used the Coryllos classification system with the following patient characteristics: Type 1 (n = 121), Type 2 (n = 155) and Types 3–4 (n = 58). Breastfeeding outcomes were subjectively reported and showed that 35% had mild improvement, followed by 14% with moderate improvement and 7% with marked improvement. Pre‐treatment baselines were mentioned but not reported.^14^ Steehler *et al*. (2012) used the Coryllos classification system with the following patient characteristics: Type 1 (n = 16), Type 2 (n = 38), Type 3 (n = 21) and Type 4 (n = 3). While baseline pre‐treatment measures were not reported, postoperative improvement in breastfeeding across Types 1–4 were 81.3%, 83.8%, 85.7% and 33.3%, respectively.^22^ Jamani *et al*. (2020) did not report any objective breastfeeding outcome measures. However, changes of HATLFF scores were reported between pre‐and post‐treatment phases. In a case series of 3 patients, baseline HATLFF scores (functional, appearance) were (7.5; 9.8; 7.8), respectively. Postoperatively, the scores changed to 13.1; 14.1; 9.1.^18^

Ferrés‐Amat *et al*. (2017) used the Coryllos classification system with the following patient characteristics: Types I & 2 (n = 33), Type III (n = 52), and Type IV (n = 3). Breastfeeding outcomes were subjective and related to maternal nipple pain based on the validated Visual Analogue Scale (VAS)^35^ (0 = no pain; 10 = severe pain) and showed improved mean scores from 5.33 to 0.81.^16^ Similarly, Ballard *et al*. (2002) reported low HATLFF functional and appearance mean scores (7.9 ± 1.86 and 4.9 ± 1.81, respectively) and demonstrated a significant reduction in pain scores (*P* < 0.001) from 6.9 ± 2.31 to 1.2 ± 1.52 after tongue‐tie release.^12^

### Studies with individually reported pre‐ and post‐treatment measures

Only one study reported changes in pre‐treatment measures and post‐treatment outcomes within an identified severity group. Ghaheri *et al*. (2016) used both the Coryllos and Kotlow classification systems and identified the following patient characteristics: Types 1 to 4 were (n = 12, n = 40, n = 76 and n = 109) for the Coryllos system, respectively; Classes I–IV were (n = 0, n = 2, n = 109 and n = 126) for the Kotlow system, respectively. Pre‐and post‐division breastfeeding difficulties were assessed using a VAS pain scale. The authors established significant postoperative improvements in VAS pain scores (*F*_(2)_ = 259.8; *P* < 0.001).^17^

### Studies with speech outcomes

Only two of the included studies reported speech related outcomes following surgical intervention. Belmehdi *et al*. (2018) described two patients classified with Kotlow’s Class II tongue‐tie who were surgically treated by a frenectomy. The authors reported excellent post‐operative results associated with improved articulation based on a subjective clinical assessment.^13^ Similarly, Kim *et al*. (2020) also used Kotlow’s classification scheme with the following patient characteristics: Class I (n = 9), Class II (n = 21), Class III (n = 7). The authors demonstrated a significant improvement in speech articulation (*P* < 0.001), based on the validated Urimal test of articulation and phonation (U‐TAP), in both treatment groups (22.44 ± 23.49 and 22.94 ± 38.69).^19^

## DISCUSSION

There is a paucity of information that facilitates the diagnosis of clinically significant tongue‐tie. This poses a challenge for the clinician to identify the correct population experiencing tongue‐tie with a clear indication for lingual frenectomy. To this end, the relationship between validated classification tools, symptomatic tongue‐tie, and frenectomy need to be examined. Evidently, only a few studies have attempted to investigate the relationship between possible contributing factors, and their results remain unclear.

A major shortcoming evident in the tongue‐tie literature is defining the severity or level of tongue‐tie, as measured during pre‐treatment assessments. The lack of agreement has made the management of tongue‐tie challenging, since not all patients with tongue‐tie will have difficulties in breastfeeding or speech. Despite the recent call for the creation of standardized tools for tongue‐tie identification,^4^ there is little or no agreement regarding a standardized clinical assessment method, resulting in minimal use of existing classification protocols to evaluate and manage tongue‐tie.^30^, ^36^ Because of the high level of heterogeneity between existing classification systems reported in the literature, comparative analysis is challenging.

According to the current findings, of the 205 generated articles, only three were RCTs while the remaining were deemed “low” on the hierarchy of evidence. The lack of high‐level evidence remains, and the general supposition regarding tongue‐tie undermines the ability to generate convincing guidelines for management.^37^ In addition, of the included articles, there were several different outcome measures applied, including U‐TAP, VAS, LATCH and IBFAT, as well as subjective reports.^12^, ^15^, ^17^, ^19^, ^21^ Because of the various types of outcome measure tools used across the included studies, a meta‐analysis was not possible. There must be greater standardization of classification systems and outcome measures with the goal of providing clinicians with standardized tools that can accurately predict patient‐specific outcomes.

All included studies reported, to some degree, improvements in outcomes. However, a major limitation across all but one study was the absence of individually reported changes in pre‐treatment severity scores and post‐treatment outcome scores. As a result, a statistical correlation could not be determined, and therefore it remains unclear whether the current classification systems provide adequate data for successful surgical management. Evidently, based on the scope of the present research question, the most used classification systems were the HATLFF (6 studies) and Coryllos (5 studies). Because it relies solely on anatomical description, there is uncertainty regarding the use of the Coryllos classification system, due to its limitation in assessing the functional restriction of tongue movement.^4^ A previous study showed no correlation between the Coryllos classification system and breastfeeding difficulties in a cohort of 200 infants.^38^ However, the HATLFF system attempts to overcomes the limitation of the Coryllos classification by including items that assess function. A major concern is the level of sophistication and time needed to complete the assessment, making routine use challenging. It can be argued that a “superior” classification system would, in theory, take into consideration both the anatomical and functional capacity of tongue‐tie.

Based on the current findings and determined by previous work, many questions remain unanswered. It has yet to be shown to what extent patients with tongue‐tie might benefit from frenectomy across all severity scores. The objective and subjective outcome measures documented in this review indicate that patients may improve function related to breastfeeding and speech. However, due to the lack of consensus regarding pre‐treatment assessment methods, diverse outcome measures, and poor study design and methodology, precise quantification of any improvement cannot be calculated. Moreover, a lack of professional education and general consensus on tools for the assessment and diagnosis of tongue‐tie has led to significant inconsistencies in practice habits. This is confounded by the wide variation in applied surgical techniques, which further complicates obtaining consensus. Nevertheless, extensive investigation is still needed in order to construct valid assessment tools that can be applied in the clinical setting.

## CONCLUSION

There is wide agreement that tongue‐tie division can improve breastfeeding and speech. However, it remains unclear to what extent severity scores on pre‐treatment assessment scales correlate with successful surgery. Therefore, the benefits of frenectomy cannot be predicted from the current classification systems. There are major inconsistencies in the literature with regards to the use of pre‐ and post‐treatment assessment tools, and a paucity of articles which report high‐level evidence. Most of these short‐comings stem from a lack of consensus between clinicians. It is recommended that future studies incorporate validated measures of the individual severity of a tongue‐tie and breastfeeding (or speech) outcome measures. Finally, additional studies with adequate designs and methodologies are needed to shed light on the association between the severity of tongue‐tie and successful surgical outcomes.

## CONFLICT OF INTEREST

The authors disclose no conflicts of interest. This research has not received any funding. All authors have viewed and agreed to the submission.
